# The effects of material type, salivary contamination and adhesive application on the performance of pit and fissure sealants

**DOI:** 10.1371/journal.pone.0352985

**Published:** 2026-07-01

**Authors:** Adem Gok, Kubra Bilge, Tuba Gok

**Affiliations:** 1 Department of Restorative Dentistry, Faculty of Dentistry, Firat University, Elazig, Turkey; 2 Department of Endodontics, Faculty of Dentistry, Firat University, Elazig, Turkey; Mersin University: Mersin Universitesi, TÜRKIYE

## Abstract

This study aimed to assess the effects of sealant material type, adhesive application and saliva contamination on the microleakage, shear bond strength (SBS), and interfacial adaptation of pit and fissure sealants. A total of 160 extracted human molars were assigned to eight groups according to sealant type (resin-based, glass ionomer-based, or flowable composite), adhesive application, and contamination condition. Microleakage and unfilled areas at the enamel–sealant interface were assessed by dye penetration and image analysis software after thermocycling. SBS was measured using a universal testing machine, and failure modes were categorized under a stereomicroscope. Interfacial adaptation was analyzed using scanning electron microscopy (SEM). Data were analyzed with Fisher’s exact, Kruskal–Wallis, Mann–Whitney U, and ANOVA tests (α = 0.05). Resin-based and flowable composite sealants exhibited significantly lower microleakage and higher SBS than glass ionomer-based materials (p < 0.05). Adhesive application enhanced SBS but slightly increased microleakage. Saliva contamination markedly reduced bond strength and increased leakage, whereas re-etching effectively restored enamel receptivity and sealing ability. SEM images revealed better adaptation and continuous margins in resin-based and flowable composites, whereas contaminated groups exhibited irregular interfaces and micro gaps. Sealant performance is strongly influenced by material viscosity, surface pretreatment, and contamination control. Re-etching after contamination restores enamel-bonding potential, while adhesive-assisted flowable composites achieve the best combination of sealing, retention, and interfacial adaptation, suggesting their clinical advantage in moisture-compromised environments.

## Introduction

Occlusal pits and fissures are highly susceptible to caries development due to their complex morphology and difficulty in cleaning [1]. Pit and fissure sealants are recognized as one of the most effective non-invasive methods to protect these sites by blocking bacterial colonization and food accumulation [1,2]. Evidence-based guidelines and systematic reviews have shown that sealed teeth exhibit a markedly lower incidence of occlusal caries than unsealed controls, with caries reduction rates ranging between 11% and 75% within two years of application [1–3].

Sealant materials are mainly classified as resin-based or glass ionomer-based [4,5]. Resin-based sealants are most commonly used due to their micromechanical retention to acid-etched enamel and superior long-term retention rates, but they are highly technique-sensitive and require strict isolation [4,6]. In contrast, glass ionomer-based sealants chemically bond to enamel, act as a fluoride reservoir, and show greater tolerance to moisture, which makes them suitable for partially erupted teeth or patients with limited cooperation [7,8]. More recently, flowable composites have been proposed as alternative sealants because of their low viscosity, favorable handling properties, and improved penetration into fissures [9,10].

To overcome the limitations associated with traditional sealants and further improve retention, recent strategies have focused on the use of adhesive systems and flowable composites [5,6,9]. Adhesive pretreatment enhances enamel penetration and bond strength while reducing microleakage, particularly when etch-and-rinse adhesive systems are used prior to fissure sealant application [5,6]. Flowable composites, owing to their low viscosity and favorable handling, have demonstrated comparable or superior retention rates to conventional resin-based sealants. Long-term clinical studies reported high retention and marginal integrity for flowable composites up to 36 months [9,11,12].

The clinical success of fissure sealants depends largely on their retention and ability to achieve a hermetic marginal seal [13,14]. Key factors influencing long-term performance include fissure morphology, adequate enamel etching, and strict isolation [13,14]. Among these, saliva contamination remains the most critical challenge, as it can occlude enamel microporosities, inhibit resin tag formation, reduce bond strength, and increase microleakage and secondary caries [15,16].

In vitro models such as shear bond strength (SBS) and dye penetration or penetration depth tests remain widely used to evaluate adhesive performance and sealing integrity. However, previous studies have reported inconsistent outcomes regarding the effectiveness of flowable composites, adhesive pretreatment, and optimal handling of saliva contamination [17,18]. To the best of our knowledge, this research is one of the first to integrate real human saliva contamination, universal adhesive pretreatment, and multiple analytical outcomes (microleakage, SBS, and scanning electron microscopy (SEM) adaptation) within a single experimental design, thereby enhancing the clinical relevance of in vitro findings. Given the conflicting findings in the literature, this study aimed to evaluate the effects of sealant type (resin-based, glass ionomer-based, and flowable composite), universal adhesive application, and saliva contamination on microleakage, SBS, and fissure adaptation. Eight experimental groups were designed under standardized in vitro conditions. The following null hypotheses were tested:

H₀1: Different fissure sealant types (resin-based, glass ionomer-based, and flowable composite) do not differ in terms of microleakage, SBS, or proportion of unfilled areas.

H₀2: The application of a universal adhesive beneath resin-based fissure sealants does not affect microleakage, SBS, or adaptation.

H₀3: Saliva contamination does not alter the microleakage, SBS, or adaptation of resin-based sealants.

H₀4: Saliva contamination does not alter the microleakage, SBS, or adaptation of glass ionomer-based sealants.

## Materials and methods

### Study design and sample size estimation

This in vitro experimental study investigated the effects of sealant type, universal adhesive application, and saliva contamination on the interfacial performance of different fissure sealants. Eight experimental groups were established according to material type and surface condition, and each group was evaluated for microleakage, SBS, and morphological adaptation under a stereo microscope, as well as for sealant–tooth interfacial adaptation under a SEM.

This study, which utilized extracted human teeth and human saliva, was reviewed and approved by the Non-Interventional Clinical Research Ethics Committee of Firat University (Approval No: 2022/09–48), in accordance with the Declaration of Helsinki. All extracted human teeth samples and saliva were accessed and prepared for research purposes after ethical approval, between 07/09/2022 and 30/12/2022. Sample size estimation for both microleakage and SBS tests was performed using data from a previous study [15]. A priori power analysis was conducted using G*Power software (v3.1.9.2; Heinrich-Heine-University, Düsseldorf, Germany), assuming a power of 95% and an α-error of 0.05. The analysis indicated that a minimum of eight teeth per group would be sufficient; however, ten teeth per group were prepared to ensure adequate statistical power and compensate for potential specimen loss. Ultimately, ten intact and valid teeth from each group were included in the final analysis.

### Specimen preparation and grouping

A total of 160 sound, caries-free human third molars extracted within the previous six months were used for the study. Teeth were examined under a stereo microscope (20×) to exclude cracks, caries, restorations, or structural anomalies. Dark, worn, or calcified teeth were excluded. Following extraction, soft tissue residues were removed with a scaler, and the teeth were cleaned with water and stored in 1% chloramine-T for 24 h at room temperature, followed by storage in saline at 4 °C. The solution was renewed every 15 days to prevent degradation.

**Fig 1:**
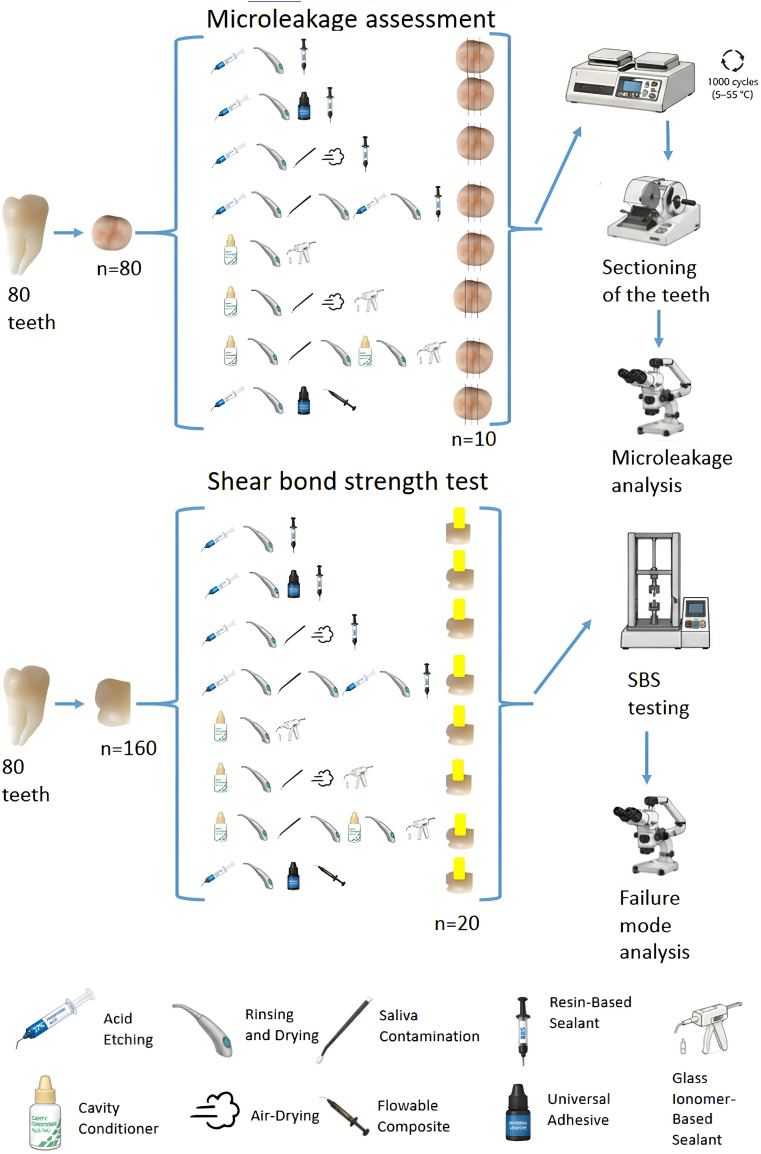
Schematic representation of the experimental design illustrating group allocation, salivary contamination protocols, microleakage assessment, and shear bond strength testing procedures performed on extracted human teeth. (**The illustration was created by the authors).**

Eighty teeth were assigned for the microleakage test and eighty for the SBS test. Teeth were randomly assigned to eight experimental groups (n = 10 per test type), based on sealant material and surface condition. The study flow diagram illustrating the grouping and experimental workflow is shown in Fig 1. The materials used and their compositions are listed in Table 1. An LED light-curing device (Valo Cordless, Ultradent, USA; 1000 mW/cm²) was used for polymerization. In the contaminated groups, fresh unstimulated human saliva collected from the operator one hour after food and beverage consumption and tooth brushing was applied to the enamel surface with a microbrush for 10 s, then either air-dried (saliva contamination 1-SC1) or rinsed and re-etched (saliva contamination 2-SC2) before sealant application [19]. The flowable composite group was included only in the sealant type comparison and was not subjected to additional experimental conditions. It was used to represent an adhesive-assisted application under ideal conditions and to serve as a reference for comparison with conventional sealant materials [10].

**Table 1:** Composition of the materials used in the present study.

Material (Brand name)	Manufacturer	Contents
Resin-based fissure sealant (Fissurit FX)	VOCO	Barium aluminum borosilicate glass, TEGDMA, UDMA, BisEMA, BisGMA, fluorosilicate glass, sodium fluoride, fumed silica, initiators, stabilizers, pigments, filler content (> 50%)
Glass ionomer-based fissure sealant (Fuji Triage)	GC Europe	Glass ionomer, aluminoﬂuorosilicate glass, polyacrylic acid, distilled water, pigment, polybase carboxylic acid
Flowable composite (Nova Compo-HF)	IMICRYL	Hydrophobic aromatic dimethacrylates, BisGMA, TEGDMA, UDMA, silanated barium glass, nano ytterbium, silanated nano highly dispersed silicon dioxide, silica zirconia and prepolymer catalysts, dl-camphorquionone, stabilizers, pigments (65–70% wt, 53–55% v/v)
Phosphoric Acid Etching Gel (Promida-Proetch)	PROMIDA	37% phosphoric acid
Cavity Conditioner (Cavity cleaning agent)	GC	20% polyacrylic acid, distilled water, aluminum chloride hydrate
Universal Adhesive (Prime & Bond Universal)	Dentsply	PENTA, 10-MDP, Active Guard^TM^ Technology crosslinker, CQ/tertiary amine, Isopropanol, water

**Abbreviations:** TEGDMA: triethylene glycol dimethacrylate, UDMA: urethane dimethacrylate, BisEMA: ethoxylated bisphenol A glycol dimethacrylate, BisGMA: bisphenol a-glycidyl methacrylate, PENTA: dipentaerythritol penta-acrylate phosphate, 10-MDP: 10-methacryloyloxydecyl dihydrogen phosphate.

### Application procedures

The experimental groups and their corresponding abbreviations were defined as follows: resin-based sealant (RBS), universal adhesive + resin-based sealant (RBS-A), saliva contamination 1 + resin-based sealant (RBS-SC1), saliva contamination 2 + resin-based sealant (RBS-SC2), glass ionomer-based sealant (GIS), saliva contamination 1 + glass ionomer-based sealant (GIS-SC1), saliva contamination 2 + glass ionomer-based sealant (GIS-SC2), and flowable composite (FC).

The procedural steps for each group were standardized as follows:

- **RBS group:** Enamel surfaces were etched with 37% phosphoric acid for 30 s, rinsed for 20 s, and air-dried for 10 s until a chalky-white appearance was observed. A light-curing resin-based fissure sealant was applied, spread for 15 s, and light-cured for 20 s.
- **RBS-A group:** The same procedure as in the RBS group was followed, except that after etching, a universal adhesive was applied with a microbrush for 15 s, gently air-thinned for 5 s, and light-cured for 10 s before applying the resin-based sealant.
- **RBS-SC1 group:** The same procedure as in the RBS group was followed, except that after etching, rinsing, and drying, the enamel surface was contaminated with freshly collected unstimulated saliva for 10 s and gently air-dried for 5 s before sealant application.
- **RBS-SC2 group:** The same procedure as in the RBS group was followed, except that after etching, rinsing, and drying, saliva was applied for 10 s, rinsed for 10 s, and the surface was re-etched for 30 s, rinsed, and dried before sealant application.
- **GIS group:** Enamel surfaces were conditioned with 20% polyacrylic acid for 10 s, rinsed for 10 s, and gently air-dried. The glass ionomer-based sealant was mixed in a capsule mixer for 10 s, applied with a capsule applier, and allowed to set for 3 min.
- **GIS-SC1 group:** The same procedure as in the GIS group was followed, except that after conditioning, the enamel surface was contaminated with saliva for 10 s and gently air-dried for 5 s before sealant placement.
- **GIS-SC2 group:** The same procedure as in the GIS group was followed, except that after conditioning, saliva was applied for 10 s, rinsed for 10 s, and the surface was reconditioned with 20% polyacrylic acid before sealant placement.
- **FC group:** Enamel surfaces were etched with 37% phosphoric acid for 30 s, rinsed for 20 s, and air-dried for 10 s. A universal adhesive was applied, air-thinned for 5 s, and light-cured for 10 s. A flowable composite was then placed, spread for 15 s, and light-cured for 20 s.

All procedures were performed by the same operator under controlled conditions to ensure standardization across groups.

### Microleakage assessment

The occlusal pits and fissures were cleaned with a pumice–water slurry and a rotating brush. Following sealant placement, all samples were stored in distilled water at 37 °C for 24 h and subjected to 1000 thermal cycles between 5 ± 2 °C and 55 ± 2 °C (30 s dwell time, 10 s transfer). This protocol was selected to provide standardized short-term artificial aging and to simulate thermal stresses associated with intraoral temperature fluctuations. Similar 1000-cycle thermocycling protocols have been used in previous in vitro fissure sealant studies evaluating microleakage, penetration ability, unfilled areas, adhesive-assisted sealant application, and saliva-contamination protocols under comparable laboratory conditions [20,21]. To prevent dye penetration from areas other than the sealant margins, the tooth apices were sealed with wax, and the crowns were coated with two layers of nail varnish, leaving a 1-mm margin around the sealant. The samples were then immersed in 0.05% crystal violet for 24 h and rinsed with distilled water for 30 s.

After dye penetration, each tooth was sectioned approximately 1 mm below the cementoenamel junction. The crowns were embedded in acrylic resin blocks compatible with the precision cutting device to ensure stability during sectioning. The acrylic-mounted specimens were then sectioned bucco-lingually at 1.5-mm intervals using a low-speed water-cooled diamond saw (Accutom-5, Struers, Denmark). Each specimen yielded approximately four sections and six surfaces, which were examined under a stereo microscope (20×). The proportions of microleakage and unfilled areas at the enamel–sealant interface were measured using image-analysis software (CamLabLite v2.1, Bresser GmbH, Rhede, Germany) by a blinded examiner. Microleakage was evaluated both categorically and quantitatively. Each group consisted of 10 teeth, and approximately 60 enamel surfaces (four sections per tooth, including mesial and distal aspects) were analyzed for microleakage and unfilled area measurements. Consequently, microleakage and unfilled-area analyses were performed at the surface level, yielding group-specific numbers of evaluable cross-sectional surfaces, as some sections were excluded due to defects, artefacts, or unclear margins.

**Fig 2:**
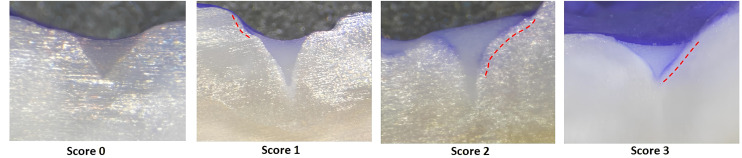
Operating microscope images (20x) of categorical microleakage. Score 0—no dye penetration, Score 1—dye penetration in the outer half of the fissure, Score 2—dye penetration in the inner half of the fissure, Score 3—dye penetration to the base of the fissure.

Categorical scoring followed a 4-point scale [22] (Fig 2)

- Score 0 = no dye penetration;
- Score 1 = dye penetration in the outer half of the fissure;
- Score 2 = dye penetration in the inner half of the fissure;
- Score 3 = dye penetration to the base of the fissure.

**Fig 3:**
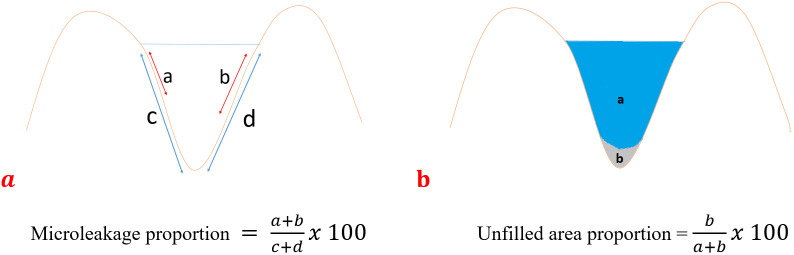
Diagrammatic depiction of the proportion of microleakage and proportion of unfilled area. a) Length of dye penetration (a + b), length of tooth–sealant interface (c + d). b) fissure/sealant area (a + b), unfilled area (b).

Quantitative (proportional) microleakage was calculated as the ratio of dye-penetrated length to the total enamel–sealant interface length (Fig 3a). The proportion of unfilled areas was calculated as the ratio of voids unfilled by the sealant to the total fissure area (Fig 3b). Fissure morphology was classified as shallow (types U, V) or deep (types Y1, Y2) [23].

### Shear bond strength (SBS) test

The buccal and lingual enamel surfaces were cleaned with a pumice–water slurry and a rotating brush. The roots were sectioned 1 mm below the cementoenamel junction using a diamond bur, and the crowns were embedded in self-curing acrylic resin with the enamel surface exposed and parallel to the upper surface. Subsequently, each tooth was cut into two halves in the mesio–distal direction using the same water-cooled diamond disc as in the microleakage test, resulting in two enamel surfaces per tooth (n = 20). Each section was embedded in self-curing acrylic resin blocks with the buccal and lingual enamel surfaces oriented upward and parallel to the testing plane to ensure proper positioning in the universal testing machine.

To obtain flat and standardized bonding areas approximately 5 mm in diameter, enamel surfaces were polished under running water with 220-, 400-, and 600-grit silicon carbide papers for 5 s each [12,15]. Cylindrical Teflon molds were used to standardize the bonded area: molds with a diameter of 1.7 mm and height of 2 mm were used for resin-based and flowable composite sealants, and molds with a diameter of 2.8 mm and height of 2 mm for glass ionomer-based sealants. Sealant materials were applied according to the same experimental procedures described earlier and light-cured as specified by the manufacturer.

After polymerization, the Teflon molds were sectioned and carefully removed. All samples were subsequently stored in distilled water at 37 °C for 24 h before testing. SBS was measured using a universal testing machine (AG-X 50 kN, Shimadzu, Kyoto, Japan) at a crosshead speed of 0.5 mm/min. A notched-edge blade was used to apply shear force at the tooth–sealant interface until failure occurred, and the SBS values were recorded in megapascals (MPa).

After debonding, the failure mode of each specimen was examined under a stereo microscope (20 × , Zumax, Suzhou, China) and categorized as:

- Adhesive failure (at the enamel–sealant interface),
- Cohesive failure (within enamel or sealant), or
- Mixed failure.

### SEM observation

Two representative specimens from each group were selected after the microleakage test to evaluate sealant penetration and interfacial adaptation. The samples were mounted on aluminum stubs, sputter-coated with gold, and examined under a SEM (EVO 40, LEO, Cambridge, UK) at 1000 × magnification. The enamel–sealant interface and the degree of adaptation to the fissure walls were qualitatively analyzed.

### Data presentation and statistical analysis

For analysis, the groups were categorized according to material type, fissure type, adhesive application, and saliva contamination. Data were analyzed using IBM SPSS Statistics software (v26.0; IBM Corp., Armonk, NY, USA). The normality of continuous data was assessed using the Shapiro–Wilk test, and the homogeneity of variances was evaluated using Levene’s test before parametric analyses. A significance level of p < 0.05 was adopted. Categorical microleakage scores were analyzed using the chi-square test or Fisher’s exact test, as appropriate according to expected cell frequencies. When multiple pairwise comparisons were required for categorical outcomes, Bonferroni correction was applied. As proportional microleakage and unfilled-area data did not meet the assumptions for parametric analysis, these variables were analyzed using the Mann–Whitney U test for two-group comparisons and the Kruskal–Wallis test for comparisons involving more than two groups. When a significant Kruskal–Wallis result was detected, Dunn’s post hoc test was used for pairwise comparisons. For SBS data, parametric tests were used because the assumptions of normality and homogeneity of variances were satisfied. Independent-samples t-tests were used for planned two-group comparisons, whereas one-way ANOVA followed by Tukey’s post hoc test was used for multiple-group comparisons. Spearman’s rank correlation analysis was performed to evaluate the association between categorical microleakage scores and proportional microleakage values. Differences between shallow and deep fissure types were analyzed using the Mann–Whitney U test.

## Results

### Categorical microleakage scoring

**Fig 4:**
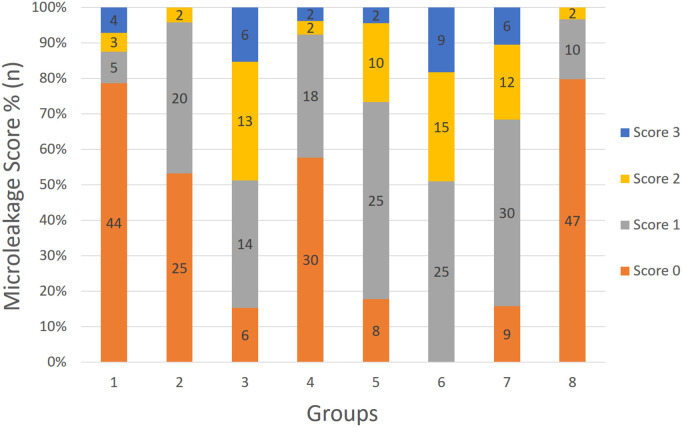
Categorical distribution of microleakage scores (% and n) for the experimental groups.

The categorical distribution of microleakage scores among the experimental groups is presented in Table 2 and Fig 4. For statistical analysis, microleakage scores 1–3 were combined and considered as ‘microleakage-positive,’ while score 0 was considered ‘microleakage-negative,’ to ensure adequate cell frequencies for chi-square and Fisher’s exact tests. A statistically significant difference was found among sealant types (p < 0.001, chi-square test). RBS and FC groups showed the highest proportion of Score 0 specimens (78.6% and 79.7%, respectively), indicating the absence of microleakage, whereas the GIS group exhibited significantly higher frequencies of Score 1–3 (82.2%), corresponding to greater dye penetration. Bonferroni-adjusted pairwise comparisons confirmed that the GIS exhibited significantly higher microleakage than both RBS and FC groups (p < 0.001). Regarding adhesive application, RBS-A group presented a higher percentage of microleakage-positive scores (46.8%) than the RBS alone group (21.4%), and this difference was statistically significant (p < 0.001, Fisher’s Exact test). In the saliva contamination (resin-based) groups, microleakage significantly increased when saliva was applied and air-dried without re-etching (RBS-SC1: 84.6% positive scores) compared with the uncontaminated control (21.4%) and the rinse-and-re-etch group (RBS-SC2: 42.3%) (p < 0.001, Fisher’s Exact test). For the glass ionomer-based sealant groups, the presence of saliva also resulted in higher microleakage scores. The GIS-SC1 group (100%) showed greater microleakage than both the control (82.2%) and the GIS-SC2 group (84.2%), with the difference being statistically significant (p < 0.001, chi-square test). Additional pairwise comparisons were performed under identical contamination conditions. Under SC1 conditions, the RBS-SC1 group showed a lower proportion of microleakage-positive samples compared to the GIS-SC1 (84.6% vs 100%, p < 0.001). Similarly, under SC2 conditions, RBS-SC2 group demonstrated significantly lower microleakage than GIS-SC2 group (42.3% vs 84.2%, p < 0.001).

**Table 2:** Distribution of categorical microleakage scores (0–3) and binary comparison (Score 0 vs Score 1–3) among sealant groups according to sealant type, adhesive application, and saliva contamination (n, %).

Comparison method	Groups	(n)	Score 0% (n)	Score 1% (n)	Score 2% (n)	Score 3% (n)	Score 0 % (n)	Score 1–3 % (n)	p value
**Sealant type**	RBS	56	78.6 (44)	8.9 (5)	5.4 (3)	7.1 (4)	78.6^A^ (44)	21.4^A^ (12)	<0.001
	GIS	45	17.8 (8)	55.6 (25)	22.2 (10)	4.4 (2)	17.8^B^ (8)	82.2^B^ (37)	
	FC	59	79.7 (47)	16.9 (10)	3.4 (2)	0 (0)	79.7^A^ (47)	20.3^A^ (12)	
**Adhesive application**	RBS	56	78.6 (44)	8.9 (5)	5.4 (3)	7.1 (4)	78.6^A^ (44)	21.4^A^ (12)	<0.001
	RBS-A	47	53.2 (25)	42.6 (20)	4.3 (2)	0 (0)	53.2^B^ (25)	46.8^B^ (22)	
**Saliva contamination (Resin-based)**	RBS	56	78.6 (44)	8.9 (5)	5.4 (3)	7.1 (4)	78.6^A^ (44)	21.4^A^ (12)	<0.001
	RBS-SC1	39	15.4 (6)	35.9 (14)	33.3 (13)	15.4 (6)	15.4^B^ (6)	84.6^B^ (33)	
	RBS-SC2	52	57.7 (30)	34.6 (18)	3.8 (2)	3.8 (2)	57.7^A^ (30)	42.3^A^ (22)	
**Saliva contamination (glass ionomer-based)**	GIS	45	17.8 (8)	55.6 (25)	22.2 (10)	4.4 (2)	17.8^A^ (8)	82.2^A^ (37)	<0.001
	GIS-SC1	49	0 (0)	51.0 (25)	30.6 (15)	18.4 (9)	0^B^ (0)	100^B^ (49)	
	GIS-SC2	57	15.8 (9)	52.6 (30)	21.1 (12)	10.5 (6)	15.8^A^ (9)	84.2^A^ (48)	

Values with the same superscript capital letter indicate no statistically significant difference (p > 0.05, post hoc pairwise comparison). Scores 1–3 were grouped as microleakage-positive for statistical analysis due to low expected frequencies in individual categories.

### Proportion of microleakage

The proportional (quantitative) analysis of microleakage is summarized in Table 3 below. Significant differences were observed among different sealant types (p < 0.001, Kruskal–Wallis test). The GIS exhibited the highest median microleakage proportion (26.43 [14.62–40.76]), whereas both the RBS and FC groups showed negligible leakage (0.00 [0.00–0.00]); these differences were statistically significant (p < 0.001, Dunn’s post hoc test). Adhesive application significantly increased microleakage in resin-based group (p = 0.040, Mann–Whitney U test). RBS-A group (0.00 [0.00–17.12]) demonstrated higher leakage values compared with the resin-based sealant applied alone (0.00 [0.00–0.00]). Regarding saliva contamination, significant differences were found among the resin-based groups (p < 0.001, Kruskal–Wallis test). The RBS-SC1 condition (saliva applied and air-dried) showed the highest microleakage (38.03 [13.66–70.73]), followed by RBS-SC2 (0.00 [0.00–16.62]) and the RBS group (0.00 [0.00–0.00]). Dunn’s post hoc test revealed that microleakage in the RBS-SC1 group was significantly greater than that in both the RBS-SC2 and control groups. For the glass ionomer-based groups, microleakage also differed significantly (p < 0.001, Kruskal–Wallis test). The GIS-SC1 group (47.46 [22.88–65.55]) exhibited the highest values, followed by GIS-SC2 (33.44 [19.94–48.20]) and the GIS (26.43 [14.62–40.76]). Dunn’s test indicated significantly higher leakage for GIS-SC1 compared with both GIS-SC2 and the GIS group. Additional pairwise comparisons were performed between materials under identical contamination conditions. Under SC1 conditions, GIS-SC1 group demonstrated significantly higher microleakage compared with RBS-SC1 group (p = 0.031). Similarly, under SC2 conditions, GIS-SC2 group showed markedly higher microleakage than RBS-SC2 group (p < 0.001).

**Table 3:** Comparison of proportional microleakage among sealant groups according to sealant type, adhesive application, and saliva contamination (Median, [Q1–Q3]).

Comparison method	Groups	(n)	Median [Q1-Q3]	*p* value
**Sealant type**	RBS	56	0.00 [0.00–0.00]^A^	<0.001
	GIS	45	26.43 [14.62–40.76]^B^	
	FC	59	0.00 [0.00–0.00]^A^	
**Adhesive application**	RBS	56	0.00 [0.00–0.00]^A^	0.040
	RBS-A	47	0.00 [0.00–17.12]^B^	
**Saliva contamination (Resin-based)**	RBS	56	0.00 [0.00–0.00]^A^	<0.001
	RBS-SC1	39	38.03 [13.66–70.73]^B^	
	RBS-SC2	52	0.00 [0.00–16.62]^A^	
**Saliva contamination (glass ionomer-based)**	GIS	45	26.43 [14.62–40.76]^A^	<0.001
	GIS-SC1	49	47.46 [32.28–65.05]^B^	
	GIS-SC2	57	33.44 [19.94–48.20]^A^	

Values with the same superscript capital letter indicate no statistically significant difference.

### Relationship between categorical microleakage scores and proportion of microleakage

A strong positive correlation was found between categorical microleakage scores and the proportion of microleakage, as determined by Spearman’s correlation test (r = 0.947, p < 0.001). Groups with higher categorical scores exhibited significantly greater proportional microleakage values. The median microleakage proportions increased progressively from Score 0 to Score 3, confirming a strong association between the categorical scoring system and the quantitative measurements of dye penetration along the enamel–sealant interface.

### Proportion of unfilled area

The proportions of unfilled areas in the fissures are presented in Table 4. A statistically significant difference was found among sealant types (p = 0.034, Kruskal–Wallis test). The GIS exhibited the highest proportion of unfilled areas (5.02 [0.00–9.91]), which was significantly higher than that of the RBS (0.00 [0.00–6.18]) (p < 0.05, Dunn’s post hoc test). The FC group (0.00 [0.00–8.59]) showed intermediate values that did not differ significantly from either the RBS or GIS groups. No significant differences were detected regarding adhesive application (p = 0.243), saliva contamination in resin-based groups (p = 0.677), or saliva contamination in glass ionomer-based groups (p = 0.806) (Mann–Whitney U and Kruskal–Wallis tests). Additional pairwise comparisons were performed between materials under identical contamination conditions. Under SC1 conditions, no statistically significant difference was observed between RBS-SC1 and GIS-SC1 groups (p = 0.205). However, under SC2 conditions, GIS-SC2 demonstrated significantly higher unfilled area values compared to RBS-SC2 group (p = 0.015).

**Table 4:** Comparison of unfilled areas in fissures among sealant groups according to sealant type, adhesive application, and saliva contamination [Median (Q1–Q3)].

Comparison method	Groups	(n)	Median[Q1-Q3]	*p* value
**Sealant type**	RBS	56	0.00 [0.00–6.18]^A^	0.034
	GIS	45	5.02 [0.00–9.91]^B^	
	FC	59	0.00 [0.00–8.59]^A, B^	
**Adhesive application**	RBS	56	0.00 [0.00–6.18]^A^	0.243
	RBS-A	47	0.00 [0.00–0.00]^A^	
**Saliva contamination (Resin-based)**	RBS	56	0.00 [0.00–6.18]^A^	0.677
	RBS-SC1	39	0.00 [0.00–8.20]^A^	
	RBS-SC2	52	0.00 [0.00–5.21]^A^	
**Saliva contamination (Glass ionomer-based)**	GIS	45	5.02 [0.00–9.91]^A^	0.806
	GIS-SC1	49	3.48 [0.00–9.94]^A^	
	GIS-SC2	57	4.51 [0.00–9.88]^A^	

Values with the same superscript capital letters indicate no statistically significant difference between groups.

### Fissure type analysis

Fissure morphology was classified as shallow (types U, V) or deep (types Y1, Y2), as shown in Table 5. The effects of fissure type on microleakage and unfilled areas are presented in Table 6. No statistically significant difference in the proportion of microleakage was found between shallow and deep fissures (p = 0.143, Mann–Whitney U test). However, the proportion of unfilled areas was significantly higher in deep fissures (8.14 [6.52–11.42]) than in shallow fissures (0.00 [0.00–0.00]) (p < 0.001, Mann–Whitney U test). This finding indicates that fissure morphology affected the completeness of sealant penetration, but not the extent of dye penetration along the enamel–sealant interface.

**Table 5:** Fissure type distribution among experimental groups (n, %). N: total number of fissure cross-sections per group.

Group		Fissure Type			
	N	U % (n)	V % (n)	Y1 % (n)	Y2 % (n)
RBS	56	17.9% (10)	30.4% (17)	35.7% (20)	16.1% (9)
RBS-A	47	23.4% (11)	29.8% (14)	31.9% (15)	14.9% (7)
RBS-SC1	39	15.4% (6)	33.3%(13)	38.5%(15)	12.8%(5)
RBS-SC2	52	17.3% (9)	25% (13)	36.5%(19)	21.2%(11)
GIS	45	11.1% (5)	22.2%(10)	55.6%(25)	11.1% (5)
GIS-SC1	49	10.2% (5)	38.8%(19)	42.9%(21)	8.2% (4)
GIS-SC2	57	12.3% (7)	40.4%(23)	33.3%(19)	14% (8)
FC	59	16.9% (10)	33.9% (20)	33.9% (20)	15.3% (9)
Total	404	15.6% (63)	31.9%(129)	38.1%(154)	14.4% (58)

**Table 6:** Comparison of proportional microleakage and unfilled area ratios between shallow and deep fissures [Median (Q1–Q3)].

Groups		Proportion of microleakage (%)	Proportion of unfilled area (%)
	n	Median, [Q1-Q3]	Median, [Q1-Q3]
Shallow fissures	192	20.18^A^ [12.71–34.88]	0.00^A^ [0.00–0.00]
Deep fissures	212	24.00^A^ [16.89–39.53]	8.14^B^ [6.52–11.42]
	404	p = 0.143	p < 0.001

Values with the same superscript capital letter indicate no statistically significant difference between groups.

### SBS analysis

The mean and standard deviation (SD) values of SBS for all groups are presented in Table 7. A statistically significant difference was found among the different sealant types (p < 0.001, one-way ANOVA). The FC group exhibited the highest SBS (25.86 ± 3.60 MPa), followed by the RBS (23.13 ± 1.88 MPa), while the GIS demonstrated the lowest value (2.71 ± 0.22 MPa). Tukey’s post hoc test confirmed that all pairwise differences among the three sealant types were statistically significant (p < 0.001). Adhesive application significantly increased SBS (p = 0.036, independent-samples t-test). The RBS-A group (24.89 ± 2.45 MPa) showed higher bond strength than the RBS alone group (23.13 ± 1.88 MPa). Regarding saliva contamination in resin-based sealant groups, a significant difference was observed (p < 0.001, one-way ANOVA). The RBS-SC1 group (7.45 ± 1.14 MPa) showed a pronounced reduction in SBS compared with both the RBS (23.13 ± 1.88 MPa) and the RBS-SC2 group (22.77 ± 2.21 MPa). Tukey’s post hoc analysis indicated no significant difference between the RBS-SC2 group and the uncontaminated control (p > 0.05). For glass ionomer-based sealant groups, SBS values also differed significantly under contamination conditions (p < 0.001, one-way ANOVA). Both GIS-SC1 (2.15 ± 0.20 MPa) and GIS-SC2 (2.36 ± 0.34 MPa) groups exhibited significantly lower bond strengths than the GIS group (2.71 ± 0.22 MPa). Additional pairwise comparisons were performed between materials under identical contamination conditions. Under SC1 conditions, RBS-SC1 group demonstrated significantly higher SBS values compared to GIS-SC1 group (p < 0.001). Similarly, under SC2 conditions, RBS-SC2 group maintained significantly higher bond strength than GIS-SC2 group (p < 0.001).

**Table 7:** Analysis of shear bond strength among different sealant groups based on sealant type, adhesive application, and saliva contamination (Mean ± SD, MPa).

Comparison method	Groups	(n)	Mean ±SD	*p* value
**Sealant type**	RBS	20	23.13 **±** 1.88^A^	<0.001
	GIS	20	2.71 **±** 0.22^B^	
	FC	20	25.86 **±** 3.60^C^	
**Adhesive application**	RBS	20	23.13 **±** 1.88^A^	0.036
	RBS-A	20	24.89 **±** 2.45^B^	
**Saliva contamination (Resin-based)**	RBS	20	23.13 **±** 1.88^A^	<0.001
	RBS-SC1	20	7.45 **±** 1.14^B^	
	RBS-SC2	20	22.77 **±** 2.21^A^	
**Saliva contamination (glass ionomer-based)**	GIS	20	2.71 **±** 0.22^A^	<0.001
	GIS-SC1	20	2.15 **±** 0.20^B^	
	GIS-SC2	20	2.36 **±** 0.34^B^	

Values with the same superscript capital letters indicate no statistically significant difference between groups.

### Failure mode analysis

**Fig 5:**
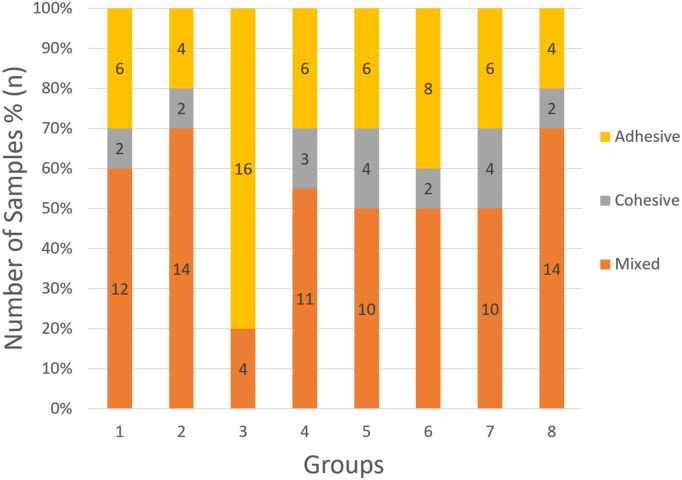
Distribution of failure modes observed after the SBS test. Adhesive failure (between the sealant and enamel), cohesive failure (within the enamel or sealant), and mixed type failure were categorized for each experimental group. (1) RBS, (2) RBS-A, (3) RBS-SC1, (4) RBS-SC2, (5) GIS, (6) GIS-SC1, (7) GIS-SC2, and (8) FC.

The distribution of failure modes among the experimental groups is shown in Fig 5. Failure patterns varied according to the sealant type and contamination condition. In both the RBS and FC groups, mixed failures were predominant, followed by adhesive failures at the enamel–sealant interface. The glass ionomer-based sealant groups exhibited a higher frequency of cohesive failures within the material, particularly under saliva-contaminated conditions. In the RBS-SC1, adhesive failures were the most frequent, reflecting the reduced bond strength observed in this condition.

### SEM observation

**Fig 6:**
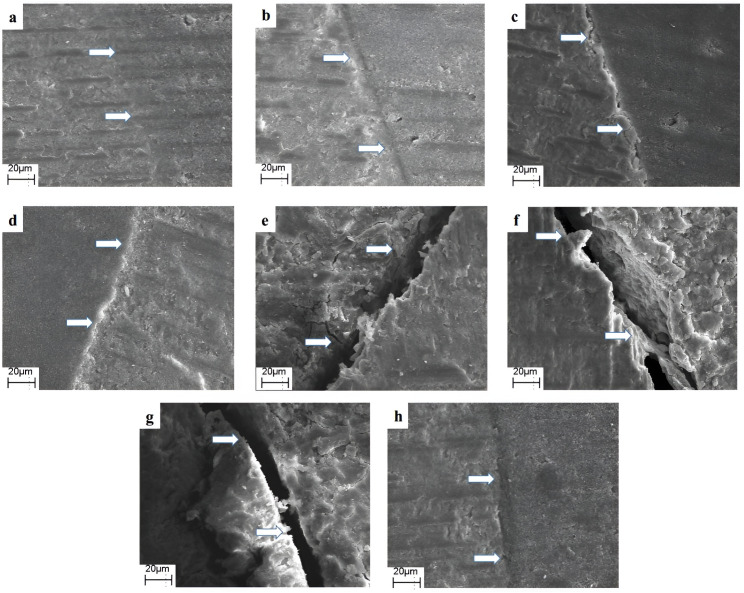
Representative SEM images (1000x) of the sealant material-enamel interface. The sealant material-enamel interfaces are indicated with arrows. **(a)** RBS, **(b)** RBS-A, **(c)** RBS-SC1, **(d)** RBS-SC2, **(e)** GIS, **(f)** GIS-SC1, **(g)** GIS-SC2, **(h)** FC.

Representative SEM micrographs of the enamel–sealant interface for each group are presented in Fig 6. The RBS and FC groups displayed intimate adaptation to the enamel surface, with continuous margins and minimal interfacial gaps. In contrast, GIS exhibited irregular interfaces and discontinuities between the material and enamel. Saliva contamination (particularly in the SC1 groups) resulted in the presence of interfacial voids and micro gaps, whereas in the SC2 groups, after re-etching or re-conditioning, the interfaces appeared more homogeneous and well adapted.

## Discussion

This in vitro study showed that sealant performance was strongly influenced by material type, adhesive pretreatment, and the management of saliva contamination. The convergence of categorical and proportional leakage data, SBS, failure modes, and SEM observations indicates that effective sealant performance is closely related to micromechanical retention and marginal sealing at the etched enamel interface.

The combined use of categorical scoring and proportional dye penetration analysis after thermocycling yielded consistent and reproducible microleakage outcomes. The strong correlation between these two measurements (r = 0.947, p < 0.001) confirms that both methods reflected the same interfacial leakage phenomenon while minimizing observer-related bias (Table 3), in line with previous findings demonstrating the reliability of dye penetration tests under thermomechanical aging conditions [22].

As retention is a key determinant of long-term clinical success, SBS serves as a practical indicator of sealant longevity. In the present study, FC exhibited the highest SBS values, followed by RBS, whereas GIS showed consistently low bond strength (Table 7). This hierarchy mirrors the corresponding microleakage and adaptation results, supporting the strong link between mechanical retention and marginal sealing integrity [24].

Fissure morphology primarily influenced the completeness of sealant penetration rather than marginal sealing. Deep (Y-type) fissures exhibited significantly higher proportions of unfilled areas compared with shallow (U/V-type) fissures (Table 6), whereas proportional microleakage did not differ between morphologies. This suggests that complex fissure geometry restricts bulk fill but does not necessarily create continuous marginal gaps. Clinically, pre-enlargement or the use of low-viscosity, adhesive-assisted sealants may help reduce voids in deep fissures without compromising marginal adaptation [14].

Saliva contamination on etched enamel disrupts micromechanical retention by forming a pellicle that blocks prism microporosities and inhibits resin tag formation [24,25]. This mechanism explains the pronounced reduction in SBS and increased leakage observed under air-dry contamination (SC1). Conversely, rinsing and re-etching effectively recreated the micro-roughened surface, restoring enamel receptivity and producing SBS values comparable to the uncontaminated control while markedly reducing leakage (Tables 2,3, and 7). These findings corroborate previous evidence that post-contamination re-etching can reverse the detrimental effects of saliva exposure [16]. Clinically, when contamination is suspected after etching, rinsing and re-etching may be considered a more reliable rescue strategy than air-drying alone. The consistently higher leakage of glass ionomer-based sealants under contaminated conditions further suggests that moisture tolerance does not necessarily compensate for weaker interfacial sealing under the present experimental conditions.

Material behavior closely followed the expected rheological and bonding characteristics. The resin-based sealant and, particularly, the flowable composite—both of low viscosity before polymerization and relying on micromechanical retention—exhibited negligible microleakage and superior SBS, as supported by SEM observations showing continuous margins and deep penetration (Fig 6). In contrast, the glass ionomer-based sealant displayed greater microleakage, more unfilled areas, and the lowest SBS, likely due to its higher viscosity and predominantly ionic bonding mechanism. These results align with previous reports demonstrating favorable sealing and mechanical performance for flowable composites and resin-based sealants compared with glass ionomer-based materials [22,26] and are further supported by systematic review evidence indicating that flowable composites can provide comparable or even superior retention [27].

Applying a universal adhesive beneath the resin-based sealant significantly increased SBS, likely due to improved enamel wettability and monomer infiltration into microporosities. However, this enhancement was accompanied by a small but significant rise in microleakage, possibly attributable to polymerization shrinkage stress or minor mismatches at the adhesive–sealant interface. From a clinical standpoint, this trade-off appears acceptable when long-term retention is prioritized, provided isolation and light-curing control are adequate. The adhesive-assisted flowable composite group achieved the highest SBS and the lowest leakage, supporting evidence that intermediate adhesives can reinforce enamel coupling and improve marginal integrity [5,28].

SEM and failure mode observations supported the quantitative findings by illustrating the interfacial consequences of material type and contamination management. Continuous resin–enamel interfaces in the resin-based and flowable composite groups were consistent with effective micromechanical retention, whereas interfacial voids and adhesive failures in contaminated specimens reflected disruption of resin-tag formation by the salivary pellicle. The predominance of cohesive or mixed failures in the RBS-A and FC groups further suggests stronger interfacial coupling, whereas cohesive failures within glass ionomer specimens likely reflected lower internal material strength rather than superior enamel bonding [24,29].

As an in vitro study, the present findings may not fully reflect the complex and dynamic intraoral environment, including continuous salivary flow, fluctuations in pH, occlusal loading, and patient-related variations in moisture control. Although thermocycling and the use of real human saliva partially simulated clinical aging and contamination, these conditions cannot fully reproduce the biological and mechanical complexity of the oral environment. The 1000-cycle thermocycling protocol used in the present study is consistent with previous in vitro fissure sealant investigations that applied similar short-term thermal aging protocols to evaluate microleakage, penetration ability, unfilled areas, and adhesive-assisted or saliva-contamination-related sealant protocols. However, this relatively limited number of thermocycles should be interpreted as a standardized short-term aging challenge rather than a simulation of long-term clinical service. Therefore, future studies using a higher number of thermocycling cycles, mechanical loading, pH cycling, long-term water storage, or clinical follow-up are needed to better evaluate the durability of the tested protocols [20,21].

Another limitation is that the flowable composite group was included as an adhesive-assisted reference material under ideal application conditions rather than as part of the full factorial saliva-contamination design. Therefore, direct comparisons of flowable composite performance under saliva-contaminated conditions or after re-etching/re-conditioning procedures could not be performed, and the findings related to this group should be interpreted within this restricted comparison framework.

Within these limitations, the present study is among the first to integrate real human saliva contamination, universal adhesive pretreatment, and multiple analytical parameters—microleakage, SBS, and SEM adaptation—within a single standardized design. This comprehensive approach may enhance the clinical relevance of in vitro sealant research and provides useful insights for optimizing sealant protocols under laboratory models of moisture-compromised conditions.

From a clinical perspective, when adequate isolation can be maintained, resin-based sealants or adhesive-assisted flowable composites may be preferred to maximize sealing and retention. If contamination occurs after etching, the present findings support rinsing and re-etching rather than air-drying alone. Glass ionomer-based sealants remain valuable for erupting molars and patients with limited cooperation due to their moisture tolerance and fluoride release; however, clinicians should anticipate lower bond strength and a greater likelihood of unfilled areas, underscoring the need for periodic monitoring and timely resealing.

## Conclusions

Within the limitations of this in vitro study, sealant performance was significantly influenced by material type, adhesive application, and saliva contamination. Flowable composite and resin-based sealants demonstrated superior sealing ability and bond strength compared to glass ionomer-based sealants. Saliva contamination adversely affected performance, whereas re-etching effectively restored bonding. These findings highlight the importance of material selection and proper contamination management in clinical practice.

## Supporting information

S1 File Dataset. (XLSX)
